# Physical and Genetic Interactions Between Uls1 and the Slx5–Slx8 SUMO-Targeted Ubiquitin Ligase

**DOI:** 10.1534/g3.113.005827

**Published:** 2013-04-01

**Authors:** Wei Tan, Zheng Wang, Gregory Prelich

**Affiliations:** Department of Genetics, Albert Einstein College of Medicine, Bronx, New York 10461

**Keywords:** Uls1, Slx5, STUbL, prion, yeast

## Abstract

The Slx5–Slx8 complex is a ubiquitin ligase that preferentially ubiquitylates SUMOylated substrates, targeting them for proteolysis. Mutations in *SLX5*, *SLX8*, and other SUMO pathway genes were previously identified in our laboratory as genomic suppressors of a point mutation (*mot1-301*) in the transcriptional regulator *MOT1*. To further understand the links between the SUMO and ubiquitin pathways, a screen was performed for high-copy suppressors of *mot1-301*, yielding three genes (*MOT3*, *MIT1*, and *ULS1*). *MOT3* and *MIT1* have characteristics of prions, and *ULS1* is believed to encode another SUMO-targeted ubiquitin ligase (STUbL) that functionally overlaps with Slx5-Slx8. Here we focus on *ULS1*, obtaining results suggesting that the relationship between *ULS1* and *SLX5* is more complex than expected. Uls1 interacted with Slx5 physically in to yeast two-hybrid and co-immunoprecipitation assays, a *uls1* mutation that blocked the interaction between Uls1 and Slx5 interfered with *ULS1* function, and genetic analyses indicated an antagonistic relationship between *ULS1* and *SLX5*. Combined, our results challenge the assumption that Uls1 and Slx5 are simply partially overlapping STUbLs and begin to illuminate a regulatory relationship between these two proteins.

The ubiquitin family consists of a group of approximately 10 structurally related but functionally distinct proteins that are conjugated to substrates as part of regulatory signal transduction pathways. As the founding member, ubiquitin is by far the best understood member of the family, with the principles and techniques that emerge from studying ubiquitin helping to guide studies of the remaining family members (Kerscher *et al.* 2006). Like ubiquitin itself, the ubiquitin family member Small Ubiquitin-like MOdifier (SUMO) has broad biological importance: the SUMO pathway is essential for viability in most eukaryotes, the components are highly conserved from yeast to humans, and more than 500 of the ∼5800 yeast proteins are posttranslationally modified by SUMO, affecting 15 major biological pathways (Bettermann *et al.* 2012; Bossis and Melchior 2006; Denison *et al.* 2005; Makhnevych *et al.* 2009; Rosonina *et al.* 2010; Shin *et al.* 2005; Wohlschlegel *et al.* 2004). The core components of the SUMO pathway responsible for the maturation, conjugation, and removal of SUMO from substrates have been extensively characterized (Desterro *et al.* 1999; Geiss-Friedlander and Melchior 2007; Johnson and Blobel 1997; Johnson *et al.* 1997; Kerscher *et al.* 2006; Li and Hochstrasser 2000), and X-ray crystallographic structures are available for most of these proteins (Capili and Lima 2007; Lois and Lima 2005; Yunus and Lima 2009), revealing details of their catalytic mechanisms.

In contrast to the extensive progress studying SUMO conjugation and de-conjugation enzymes, less is known about regulators and downstream effectors of SUMOylation. SUMOylation can have different effects on its target proteins, mediated by disrupting or creating protein-protein interactions. These altered interactions result in different biological outcomes for different substrates, including changes in cellular localization and blocking or stimulating proteolytic degradation (Huang *et al.* 2003; Lallemand-Breitenbach *et al.* 2008; Lin *et al.* 2006). The differential downstream effects are very likely mediated by recognition of the SUMOylated substrate by different SUMO-binding effector proteins. One such SUMO-binding effector that begins to account for the differential effects of SUMO is Slx5-Slx8. Slx5-Slx8 is a heterodimeric ubiquitin E3 ligase that preferentially targets selective SUMO conjugates for ubiquitylation (Mullen and Brill 2008; Prudden *et al.* 2007; Tatham *et al.* 2008; Uzunova *et al.* 2007; Xie *et al.* 2007); Slx8 is the active ubiquitin E3 ligase, and it is recruited to SUMOylated substrates by its Slx5 partner, which possesses several SUMO-interacting motifs (SIMs). Because the Slx5-Slx8 complex and its *Schizosaccharomyces pombe*, *Drosophila*, and human orthologs preferentially ubiquitylate SUMOylated substrates, they have been termed SUMO-Targeted Ubiquitin Ligases (STUbLs). This finding was unexpected because SUMO was proposed to compete with ubiquitin for some substrates (Desterro *et al.* 1998), but the existence of STUbLs demonstrated that SUMOylation can actually stimulate ubiquitylation of some proteins. Slx5-Slx8 also can target substrates via a SUMO-independent mechanism (Xie *et al.* 2010), but because of the notorious difficulty in identifying E3 substrates, it is currently unclear how many of its substrates are SUMO-dependent *vs.* SUMO-independent.

The finding that Slx5-Slx8 and its orthologs are STUbLs has raised the issue of whether other STUbLs exist. Rad18 targets PCNA for ubiquitylation through its intrinsic SUMO-binding activity and, thus, has the properties of a STUbL (Parker and Ulrich 2012). Another candidate STUbL is Uls1 (Zhang and Buchman 1997). *ULS1/RIS1* is a nonessential gene, encoding a protein of 1619 amino acids that contains multiple SIMs, a RING domain, and a Swi2/Snf2-like ATPase domain. The combination of SIMs and RING domain in Uls1 suggests possible STUbL activity, and in support of this idea, Uls1 binds to SUMO and SUMOylated proteins (Arnett *et al.* 2008; Meednu *et al.* 2008; Shirai and Mizuta 2008; Uzunova *et al.* 2007), it interacts with the Ubc4 ubiquitin E2 in pull-down assays (Uzunova *et al.* 2007), *uls1Δ* displays synthetic growth defects with *slx5Δ* or *slx8Δ* (Pan *et al*., 2006), and in *uls1Δ slx5Δ* double mutants, SUMO conjugates were reported to accumulate to a greater extent than in *slx5Δ* or *uls1Δ* single mutants (Uzunova *et al.* 2007). Combined, this information suggested that Uls1 might be another STUbL with some functional overlap with Slx5-Slx8, although their specific relationship remains unknown. Importantly, ubiquitin E3 activity has not been reported for Uls1 to date, and thus, other possibilities need to be considered.

Our laboratory has been using a genetic approach to investigate the SUMO pathway. We previously found that a mutation in *MOT1*, which encodes an essential transcriptional regulator that removes TATA-binding protein from DNA (Auble *et al.* 1994), is extremely sensitive to perturbation of the SUMO pathway. Ninety-seven percent of mutations that suppressed *mot1-301* expression were in genes that encode components of the SUMO pathway (Wang *et al.* 2006), and mutations in every step of the SUMO pathway suppressed *mot1-301*. This selection, thus, is highly sensitive and extremely selective to defects in SUMOylation. Mot1-301 is an unstable protein due to its SUMO-, ubiquitin-, and proteosome-dependent degradation, and mutations in the SUMO pathway, the Slx5-Slx8 STUbL, the ubiquitin E2 Ubc4, or in K101 and K109, the presumed SUMOylation sites of Mot1-301, dramatically stabilize the protein, accounting for the suppression phenotype (Wang and Prelich 2009). Here we continue to take advantage of this system, using an overexpression strategy in an attempt to uncover additional components or regulators of the SUMO pathway. We report physical interactions and additional genetic interactions between Uls1 and Slx5-Slx8. Furthermore, we show that this interaction is important for Uls1 function, as loss of this interaction results in physiological deficiencies. Additionally, we report that Slx5 is SUMOylated and that its SUMOylation is reduced by *uls1Δ*. These data are most consistent with a regulatory relationship between these two proteins rather than the current model in which they act as semiredundant STUbLs.

## Materials and Methods

### Strains, plasmids, and media

*Saccharomyces cerevisiae* strains used in this study are listed in Table 1. All plasmids used in this study are listed in Table 2. All media used, including rich medium (YPD) and synthetic complete drop-out medium (for example, SC–Ura) were made as described previously (Rose *et al.* 1990). SC+Gal plates were synthetic complete (SC) medium containing 2% galactose and 1 μg/ml antimycin A. Standard genetic methods for mating, sporulation, transformation, and tetrad analysis were used throughout this study (Rose *et al.* 1990).

**Table 1:** *S*. *cerevisiae* strains used in this study

Strain	Genotype
GY285	*MATα leu2Δ1 ura3-52*
GY2280	*MATα his4-912δ lys2-128δ suc2Δuas(-1900/-390) ura3-52 trp1Δ63 leu2Δ1 uls1Δ*::*KANMX*
GY2296	*MATα his4-912δ lys2-128δ suc2Δuas(-1900/-390) ura3-52 trp1Δ63 leu2Δ1 uls1Δ*::*TRP1 mot1-301*
OY844	*MAT****a*** *his4-912δ suc2Δuas(-1900/-390) trp1Δ63 leu2Δ ura3-52 mot1-301 hsp104Δ*::*KAN*
OY843	*MATα his4-912δ his3Δ200 trp1Δ63 leu2Δ ura3-52 mot1-301 hsp104 Δ*::*KAN*
ZY48	*MATα his4-912δ lys2-128δ suc2Δuas(-1900/-390) ura3-52 leu2Δ1 slx5Δ*::*URA3 mot1-301*
ZY142	*MATα his4-912δ lys2-128δ suc2Δuas(-1900/-390) leu2Δ1 trp1Δ63 mot1-301*
ZY356	*MATα his4-912δ lys2-128δ suc2Δuas(-1900/-390) ura3-52 trp1Δ63 leu2Δ1 ade8 mot1-301*
ZY528	*MATα his4-912δ lys2-128δ suc2Δuas(-1900/-390) ura3-52 trp1Δ63 leu2Δ1 K.I.TRP1-SLX5p-TAP-SLX5*
ZY616	*MATα his4-912δ lys2-128δ suc2Δuas(-1900/-390) ura3-52 trp1Δ63 leu2Δ1 mot1-301-3HA-KAN*

### Screening for high-copy suppressors

To screen the systematic YGPM library (Jones *et al.* 2008) (pool and 96-well plates), a 100-ml GY2150 (*mot1-301*/*mot1-301* diploid) culture was grown at 30°C to 2 × 10^7^ cells/ml. Cells were harvested and resuspended in water to a density of 4 × 10^9^ cells/ml. Aliquots containing approximately 4 × 10^7^ cells were placed into each well of a 96-well plate, pelleted at 3500 rpm for 10 min, and resuspended in 50 μl of transformation buffer (0.3 M LiOAc, 0.8 mg/ml salmon sperm carrier DNA). Ninety-six nanograms of each library plasmid DNA were added, cells were mixed with a multitube vortexer for 2 min, and 100 μl of 50% PEG (product code P3640-500G; Sigma) was added to each well. Cells were mixed for an additional 5 min. After a 2-hr incubation at 42°C, cells were pelleted, resuspended in water, and spotted to SC-leucine plates. Transformants were resuspended in water, plated on selective plates, and grown at 30°C for 2–4 days, and pinned to selective SC+Gal-Leu plates at 37°. Three plasmids were obtained, and by subcloning the responsible genes, we identified *MOT1*, *ULS1*, and *MIT1*. Because the known high-copy suppressors *UBA2*, *UBC9*, *SMT3*, and *ULP2* did not emerge from this screen, a random genomic library (Yoshihisa and Anraku 1989) was transformed into ZY142 (*mot1-301* haploid), screening for Ts^+^ and Gal^+^ phenotypes. From 9000 transformants examined, *MOT1* (1×), *MOT3* (2×), and *ULS1* (6×) were identified as high-copy suppressors.

**Table 2:** Plasmids used in this study

Plasmid	Genotype
pTW10	AMP 2μ *LEU2 MOT3*
pTW11	AMP 2μ *LEU2 ULS1*
pTW13	AMP 2μ *URA3 MOT3*
pTW24	AMP 2μ *LEU2 MIT1*
pTW32	AMP 2μ *LEU2 GAL4-AD-ULS1*
pTW48	AMP 2μ *LEU2 uls1Δ370-373*
pTW49	AMP 2μ *LEU2 GAL4-AD-uls1Δ531-897*
pTW76	AMP CEN *LEU2 HA-ULS1*
pTW78	AMP 2μ *LEU2 HA-uls1Δ370-373*
pTW94	AMP 2μ *LEU2 HA-uls1-D1108A*,*E1109A*
pTW96	AMP 2μ *LEU2 uls1-C1385S*
pTW104	AMP 2μ *LEU2 uls1-D1108A*,*E1109A*
pTW105	AMP 2μ *LEU2 HA-uls1-C1385S*
pTW118	AMP CEN *LEU2 3HA-SLX5*
pTW120	AMP 2μ *LEU2 3HA-SLX5*
pTW125	AMP CEN *LEU2 3HA-SLX5-K31R*
pTW129	AMP CEN *LEU2 3HA-SLX5-K465R*,*K473R*
pTW131	AMP 2μ *LEU2 3HA-SLX5-K465R*,*K473R*
pTW133	AMP CEN *LEU2 SLX5-GFP*
pTW135	AMP CEN *LEU2 3HA-SLX5-K31R*,*K465R*,*K473R*
pTW137	AMP 2μ *LEU2 3HA-SLX5-K31R*,*K465R*,*K473R*
pTW143	AMP 2μ *LEU2 uls1-ΔRING(1328-1386)*
pTW145	AMP 2μ *LEU2 HA-uls1-ΔRING(1328-1386)*
pTW147	AMP 2μ *LEU2 GAL4-AD-uls1(531-897)*
pTW149	AMP 2μ *LEU2 GAL4-AD-uls1(554-955)*
pTW151	AMP CEN *LEU2 HA-uls1Δ370-373*
pTW153	AMP CEN *LEU2 uls1-ΔRING(1328-1386)*
pTW155	AMP CEN *LEU2 HA-uls1-C1385S*
pTW163	AMP CEN *LEU2 HA-uls1Δ531-897*
pTW167	AMP CEN *LEU2 HA-uls1-D1108A*,*E1109A*

### Yeast two-hybrid assays

pGBKT7- and pGADT7-based plasmids containing Gal4BD or Gal4AD fused to *SLX5*, *SLX8*, *UBC9*, and *SMT3* were described previously (Ii *et al*. 2007). *ULS1* and its derivatives were cloned into pGADT7 or pGBKT7 by standard PCR-based cloning. Combinations of plasmids were transformed into the yeast two-hybrid reporter strain PJ69-4A (James *et al.* 1996) and selected on SC plates lacking leucine and tryptophan. Positive interactions were detected on SC plates lacking leucine, tryptophan, and adenine.

### Co-immunoprecipitation

For immunoprecipitation and Western analyses Uls1 was tagged at the N terminus with a single HA tag. HA-Uls1 was functional in all assays tested. Strains were incubated in selective medium and grown to late log phase. Cells were harvested and resuspended in pre-cooled RNP buffer (20 mM pH 7.4 HEPES, 100 mM NaCl, 0.1% NP-40, 1 mM PMSF, leupeptin/pepstatin [1mg/ml in DMSO], aprotinin [1mg/ml], and Roche Pro inhibitor). The resuspended culture was frozen in liquid nitrogen, collected into a pre-chilled 50-ml tube, and lysed with a Retsch MM301 ball mill. The cryogenic cell powder was centrifuged at 4°C and 14,000 rpm **(**Sorvall RC 6 Plus centrifuge, F13s-14×50cy rotor) for 30 min, and the supernatant stored at −80°. To collect the protein extract, we added stored supernatant to 0.6× (weight/volume) of RNP buffer. The lysed sample was centrifuged at 18,000 rpm for 30 min at 4°C, and supernatant was collected. A 0.5-mg sample was incubated with either 100 μl of 50% IgG beads (catalog no. 17-0969-01; GE), 30 μl of HA beads (catalog no. A-2095; Sigma) or 40 μl of FLAG beads (catalog no. A-2220; Sigma) for 1.5 hr at 4°C, washed three times, and loaded onto an SDS polyacrylamide gel for detection by Western blotting.

### RT PCR assays

To isolate RNA, 10 ml of cell culture (1 × 10^7^ cells/ml) was centrifuged, washed with 1 ml of H_2_O, and resuspended in 0.2 ml of RNA breaking buffer (0.5 M NaCl, 0.2 M Tris-HCl [pH 7.6], 0.01 M EDTA, 1% SDS). Two hundred fifty microliters of washed glass beads and 0.2 ml of phenol:CHCl_3_ (equilibrated in RNA breaking buffer without SDS) were added. After samples were vortexed for 2 min, 0.3 ml more RNA breaking buffer and 0.3 ml more phenol:CHCl3 were added. The aqueous phase was collected after centrifugation and extracted with 0.3 ml of phenol:CHCl_3_. The aqueous phase was collected and mixed with ethanol. After 0.5 hr at −70°C, the pellet was harvested by centrifugation, washed with 70% ethanol, and dried in a Speed Vac. The final RNA product was dissolved in 100 μl of sterile water. Two micrograms of RNA was used to quantify the transcription level of the targeted gene with SYBR Green RT-PCR reagent kit (catalog no. 4310179; Life Technologies) and optimized primer concentrations. Primers used to detect *MOT1* were GO1952 (5′TCTCTTCGACCCCGATAACG) and GO1953 (5′TGCTTGGGAATCGCCATT), and *G6PDH* was detected using GO1954 (5′GATGTCCCACACCGTCTCTTC) and GO1955 (5′GGCCACCGTCAAAAAAACG).

### Assays of protein half-life

Yeast cultures were grown overnight at 30°C to log phase in selective medium to maintain plasmids carrying the 3HA-tagged *mot1-301* alleles. To start the chase, 1 ml of culture was first collected at time zero in an Eppendorf tube pre-loaded with 10 μl of 10% sodium azide. Cells were then pelleted and frozen on dry ice. Cycloheximide (catalog no. C7698; Sigma) was added to the remainder of the culture to a final concentration of 0.5 mg/ml, and 1-ml samples were collected every 10 or 20 min in tubes containing sodium azide and frozen on dry ice. Crude extracts were prepared by the postalkaline extraction method (Kushnirov 2000). Ten microliters of supernatant were loaded for SDS-PAGE, followed by Western blotting analysis using anti-HA antibody (code SC-7392; Santa Cruz Biotechnology) to detect Mot1 or anti-G6PDH (code A9521; Sigma) to detect G6PDH as a loading control.

## Results

### Identification of high-copy suppressors of *mot1-301*

**Figure 1:**
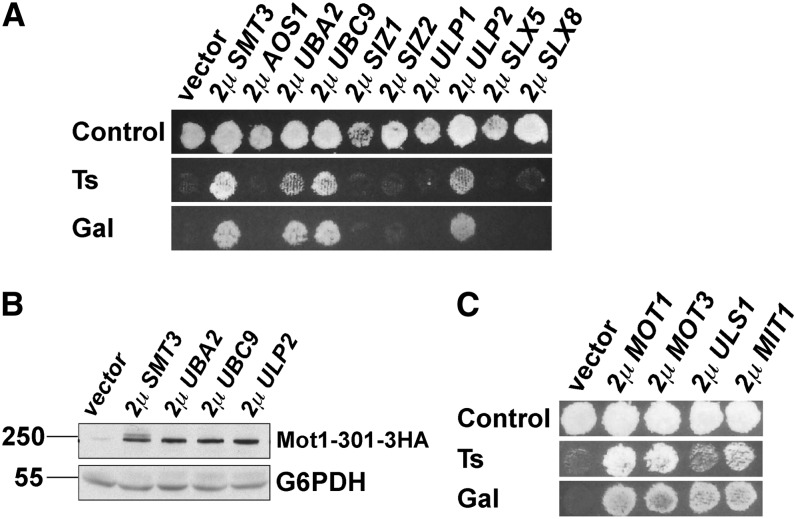
Identification of high-copy suppressors of *mot1-301*. (A) 2µ plasmids with the indicated known SUMO pathway genes were transformed into ZY142 (*mot1-301*), and transformants were replica plated to test the indicated phenotypes. Overexpression of some, but not all, known SUMO pathway components suppressed *mot1-301*. (B) The indicated 2µ plasmids were transformed into strain ZY616 (*mot1-301-3HA*) and levels of Mot1-301 detected by Western blot analysis of crude extracts. All four suppressing plasmids increased the Mot1-301 protein level relative to that of the empty vector transformant. G6PDH served as the loading control. (C) 2µ *ULS1*, *MOT3*, and *MIT1* suppressed the Ts^-^ and Gal^-^ phenotypes of *mot1-301*. Ts phenotype refers to growth at 38.5°C. Control refers to SC-Leu plates at 30°C.

We previously found that selecting for mutations that suppress *mot1-301* is a remarkably sensitive method to identify mutations in the SUMO pathway (Wang *et al.* 2006). Mutations that affect the SUMO pathway, the Slx5-Slx8 STUbL, or the Ubc4 ubiquitin E2 stabilize Mot1-301, thus suppressing the *mot1-301* phenotypes (Wang and Prelich 2009). Because this system is so specific for detecting perturbation of the SUMO-STUbL pathway, we reasoned that variations of this selection might identify additional SUMO pathway components or regulators. To determine whether an overexpression strategy might be productive, we began by testing whether directed overexpression of known SUMO pathway components suppress *mot1-301*. Indeed, overexpression of *SMT3* (SUMO), *UBA2* (E1 subunit), *UBC9* (E2), and *ULP2* (isopeptidase) all suppressed the *mot1-301* temperature-sensitive (Ts^-^) growth and Gal^-^ phenotypes (Figure 1A), and overexpression of those genes increased the Mot1-301 protein level (Figure 1B), suggesting that unknown components or regulators of the pathway might be revealed by selecting for additional high-copy suppressors of *mot1-301*. Somewhat unexpectedly, overexpression of the other known SUMO pathway genes, *AOS1* (E1 subunit), *SIZ1* (E3), *SIZ2* (E3), *ULP1* (isopeptidase), and *SLX5* and *SLX8* (STUbL subunits), did not suppress *mot1-301* (see *Discussion*).

**Figure 2:**
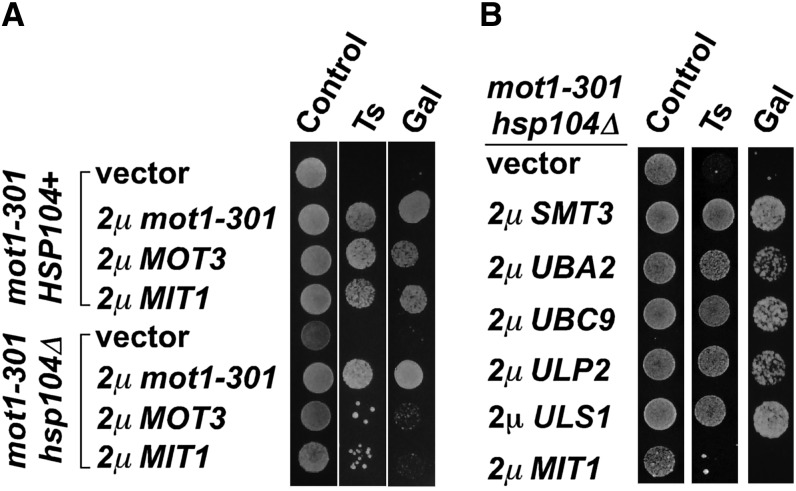
*HSP104* is required for the high-copy phenotype of *MOT3* and *MIT1* but not for SUMO pathway-mediated suppression of *mot1-301*. (A) The indicated 2µ plasmids including empty pRS425 vector were introduced into ZY142 (*mot1-301 HSP104*^+^) and OY844 (*mot1-301 hsp104*Δ) strains, and dilutions of transformants were spotted to test the Ts and Gal phenotypes. (B) Known SUMO pathway genes that suppress *mot1-301* were overexpressed in OY844 (*mot1-301 hsp104*Δ), and the indicated phenotypes were tested by spotting. Control refers to SC-Leu plates at 30°C.

Having found that overexpression of known SUMO pathway genes can suppress *mot1-301*, we screened two 2μ plasmid libraries (Jones *et al.* 2008) (Yoshihisa and Anraku 1989) for high-copy suppressors of the *mot1-301* Ts^−^ and Gal^−^ phenotypes. *MOT1* and three additional genes were identified in these screens: *ULS1*, *MOT3*, and *MIT1* (Figure 1C). *ULS1* has been functionally linked with the SUMO pathway, whereas *MOT3* and *MIT1* each encode transcriptional regulators that contain prion-like domains (Alberti *et al.* 2009). To determine whether overexpression of *MOT3* and *MIT1* suppressed *mot1-301* via prion formation and, if so, whether *ULS1* shared that property, *MOT3*, *MIT1*, and *ULS1* were overexpressed in the *mot1-301 HSP104^+^* and *mot1-301 hsp104Δ* strains. *HSP104* is required for prion formation, and thus, reversal of the *MOT3* and *MIT1* high-copy phenotypes would be indicative of involvement of prions. Indeed, 2μ *MOT3* and *MIT1* were unable to suppress *mot1-301* in the *hsp104Δ* strain (Figure 2A), whereas suppression by overexpression of *mot1-301* itself was unaffected by *hsp104Δ*. The requirement for *HSP104* was specific, as *hsp104Δ* did not reverse the high-copy phenotypes of 2μ *ULS1*, *SMT3*, *UBA2*, *UBC9*, or *ULP2* (Figure 2B). This result indicated that overexpression of *ULS1* suppresses *mot1-301* by a mechanism that is more related to the SUMO pathway and distinct from the prion-like genes *MOT3* and *MIT1*. The remainder of this report focuses on *ULS1*.

### Insights Into the suppression mechanism of 2μ *ULS1*

**Figure 3:**
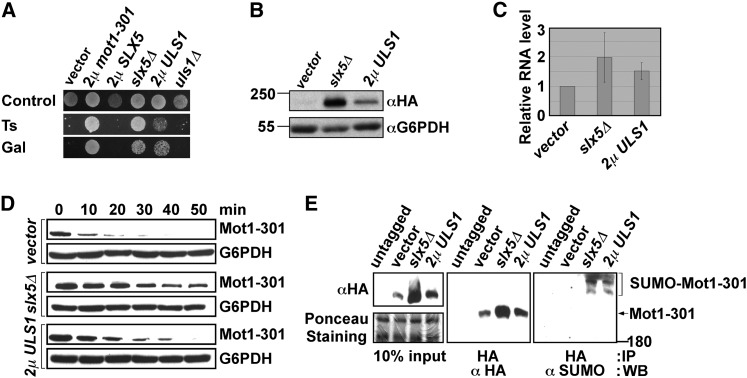
2µ *ULS1* and *slx5*Δ share similar mechanism of *mot1-301* suppression. (A) The indicated 2µ plasmids or deletions were introduced into a *mot1-301* background and spotted onto selective plates to test the Ts and Gal phenotypes. (B) Empty vector or a 2µ *ULS1* plasmid or *slx5*Δ were introduced into a *mot1-301-3HA* strain, and the protein level of Mot1-301 was assayed by Western blotting. Mot1-301 protein level increased in the 2µ *ULS1* strain, although not as much as in the *slx5Δ* control. G6PDH served as loading control. (C) The transcription level of *mot1-301* was assayed by Real-Time PCR in ZY142 (*mot1-301*), ZY613 (*mot1-301 slx5*Δ), and ZY142 transformed with 2µ *ULS1*. (D) Mot1-301 stability was examined in ZY142 (*mot1-301*), ZY613 (*mot1-301 slx5*Δ), and ZY142 transformed with 2µ *ULS1* by Western blotting during a cycloheximide chase. (E) The levels of Mot1-301 SUMOylation were assayed in ZY142 (*mot1-301*), ZY613 (*mot1-301 slx5*Δ), and ZY142 transformed with 2µ *ULS1*. Proteins were subjected to immunoprecipitation (IP) and Western blotting (WB) using the indicated antibodies. The α-HA blot on the left shows the level of Mot1-301 protein in crude extracts, and Ponceau staining of the filter shows equivalent transfer of proteins across the membrane.

We were intrigued by the ability of 2μ *ULS1* to suppress *mot1-301* because although *ULS1* has been functionally linked with the SUMO pathway, it is proposed to be another STUbL that functionally overlaps with Slx5-Slx8. We therefore tested whether deletion of *ULS1* suppressed *mot1-301*. Although *slx5Δ* suppressed *mot1-301*, *uls1Δ* did not, and conversely, 2μ *ULS1* suppressed *mot1-301* but 2μ *SLX5* did not (Figure 3A). Thus, *ULS1* and *SLX5* displayed opposing patterns of suppression. The mechanism by which SUMO pathway mutations suppress *mot1-301* is understood: the Mot1-301 protein becomes SUMOylated, presumably as part of a quality control surveillance mechanism (Wang and Prelich 2009), which results in recruitment of the Slx5-Slx8 STUbL, followed by ubiquitylation and proteosome-mediated degradation of Mot1-301. SUMO pathway defects thereby increase Mot1-301 stability and steady-state protein levels, with SUMOylated Mot1-301 accumulating in *slx5Δ* strains. We examined whether overexpression of *ULS1* had the same effects. As expected, 2μ *ULS1* increased the Mot1-301 protein level (Figure 3B), and no significant change in *mot1-301* transcription was detected by RT-PCR (Figure 3C). The stability of Mot1-301 was examined using a cycloheximide chase protocol, revealing an increase in Mot1-301 protein stability when *ULS1* was overexpressed (Figure 3D). Finally, 2μ *ULS1* increased the level of Mot1-301 SUMOylation similar to that of the *slx5Δ* control (Figure 3E). Thus, by these criteria, the overexpression of *ULS1* mimics effects caused by deletion of *SLX5*, with 2μ *ULS1* causing slightly weaker effects than *slx5Δ*.

**Figure 4:**
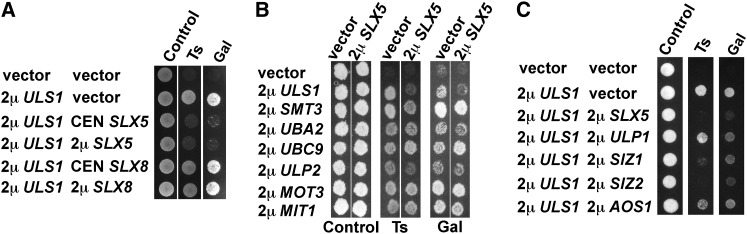
*ULS1* is genetically antagonistic to SLX5. (A) Overexpression of *SLX5* reversed the 2µ *ULS1* suppression of *mot1-301*. Combinations of the indicated plasmids were introduced into strain ZY142 (*mot1-301*), and transformants were spotted onto selective plates to test the Ts and Gal phenotypes. (B) Empty vector or 2µ *SLX5* was transformed into strain ZY356 (*mot1-301*) overexpressing the indicated genes, and the phenotypes of double transformants were tested. Relative to the empty vector transformants, overexpression of *SLX5* only reversed the phenotypes of 2µ *ULS1* not that of the other high-copy suppressors. (C) Empty vector or 2µ *ULS1* was transformed into strain ZY356 (*mot1-301*) overexpressing the indicated genes, and the phenotypes of double transformants were tested. A range of suppression is observed, with 2µ *SLX5* and 2µ *SIZ2* showing the strongest effect.

A plausible genetic interpretation for the similarities between *slx5Δ* and 2μ *ULS1* was that overexpression of *ULS1* counteracted or interfered with the function of the Slx5-Slx8 STUbL. If this model was true, then overexpressing *SLX5* or *SLX8* might reverse the 2μ *ULS1* phenotype. A *mot1-301* strain containing a 2μ *ULS1* plasmid therefore was transformed with 2μ (high-copy number) and CEN (low-copy number) plasmids containing *SLX5* or *SLX8*. Indeed, the 2μ *ULS1* phenotype was reversed by 2μ *SLX5* but not by 2μ *SLX8*, and surprisingly, even a CEN *SLX5* plasmid reversed the 2μ *ULS1* phenotype (Figure 4A). This result supported the idea that 2μ *ULS1* might suppress *mot1-301* by inhibiting or interfering with *SLX5*. Because Uls1 and Slx5 both interact physically with the ubiquitin E2 Ubc4 (Uzunova *et al.* 2007), we tested whether overexpression of *UBC4* reversed the suppression of *mot1-301* by 2μ *ULS1*. The suppression caused by 2μ *ULS1* was not abolished (data not shown), suggesting that 2μ *ULS1* does not suppress *mot1-301* by titrating Ubc4 away from Slx5. To determine whether suppression by 2μ *SLX5* was specific for *ULS1* or instead was a more general phenomenon, we tested whether overexpression of *SLX5* was able to reverse the phenotypes of other high-copy suppressors of *mot1-301*. 2μ *SLX5* reversed the Ts^+^ and Gal^+^ phenotypes caused by 2μ *ULS1* but not those of 2μ *SMT3*, *UBA2*, *UBC9*, *ULP2*, *MOT3*, or *MIT1* (Figure 4B). In another test of specificity, the SUMO pathway genes whose overexpression does not suppress *mot1-301* were tested for the ability to reverse the 2μ *ULS1* phenotype. Overexpression of those SUMO pathway genes reversed the 2μ *ULS1* phenotype to various extents, with 2μ *SLX5*, *SIZ1*, and *SIZ2* having the strongest effect (Figure 4C). Together these results suggest that *ULS1* opposes or antagonizes *SLX5*.

### Physical interaction between Uls1 and Slx5

**Figure 5:**
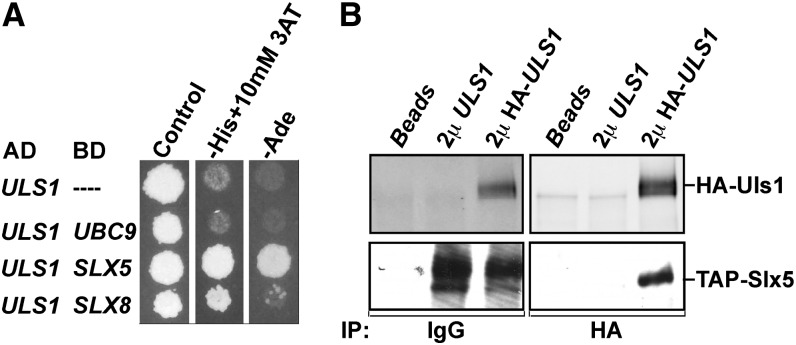
Physical interactions between Uls1 and Slx5-Slx8. (A) Plasmids expressing the indicated Gal4 activation domain (AD) and Gal4 DNA-binding domain (BD) fusions we retransformed into the yeast two-hybrid reporter strain PJ69-4A (James *et al.* 1996). Transformants were replica plated to test the two-hybrid reporter His and Ade phenotypes. C-His plates contained 10 mM 3AT to reduce nonspecific background growth. A strong interaction was detected between Uls1 and Slx5 and a much weaker interaction between Uls1 and Slx8. (B) Co-IP confirmed the physical interaction between Uls1 and Slx5. Plasmids expressing untagged or HA-tagged Uls1 were introduced into strain ZY528 (genomic TAP-Slx5). An overnight culture was harvested, cell extracts were prepared, and the proteins were subjected to immunoprecipitation (IP) as indicated, probing with either anti-HA (top panels) or anti-TAP (bottom panels) antibodies. IgG beads were used to immunoprecipitate TAP-Slx5.

Prompted by the genetic interactions between Slx5 and Uls1, we next determined whether we could detect any physical interactions between these two proteins. We first assayed for physical interactions between Uls1 and Slx5-Slx8 by using the yeast two-hybrid system. Gal4AD-Uls1 was positive with Gal4BD-Slx5 in the two-hybrid system but not with empty Gal4BD or Gal4BD-Ubc9 controls (Figure 5A). A much weaker interaction between AD-Uls1 and BD-Slx8 also was observed. Co-immunoprecipitation (Co-IP) assays were performed to further test for physical interactions between Uls1 and Slx5. Tagged versions of Uls1 and Slx5 co-immunoprecipitated regardless of which protein was immunoprecipitated (Figure 5B), whereas no Uls1-Slx8 interaction was detectable by co-IP (data not shown). These results indicated that a physical interaction occurs between Uls1 and Slx5, although from these assays, we cannot distinguish whether the interaction is direct or requires intermediates.

**Figure 6:**
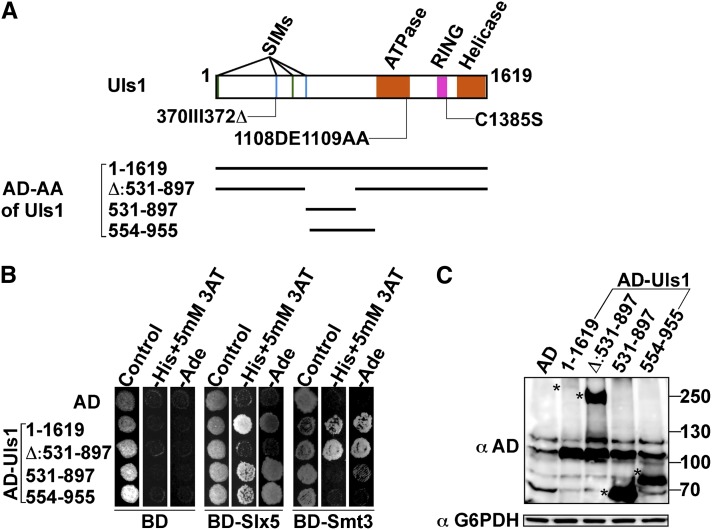
Identifying the region of Uls1 that interacts with Slx5. (A) A schematic illustration of the Uls1 domain architecture is displayed on top, with mutations in the SIM, ATPase, and RING domains shown below the protein. Uls1-AD two-hybrid derivatives are on the bottom. (B) Interactions of different Uls1-AD fragments were tested in the yeast two-hybrid system. Full-length Gal4AD-Uls1 (1–1619) and its indicated derivatives were retransformed into the yeast two-hybrid reporter strain PJ69-4A expressing binding domain (BD) only, BD-Slx5, or BD-Smt3 (SUMO). Transformants were selected and replica plated to test their His and Ade phenotypes, which are indicative of activation of the two-hybrid reporters. C-His plates contained 10 mM 3AT to reduce background signal. (C) Gal4AD-Uls1 and its derivatives were assayed for their expression level by Western blotting. The asterisks indicate migration of the respective fusion proteins.

To gain insight into the physical interaction between Uls1 and Slx5, we next examined the domains of Uls1 that were required for this interaction. In particular, because both Uls1 and Slx5 contain SIMs, we wanted to determine whether the interaction was mediated by the SIMs and SUMO. Different *ULS1* fragments were constructed into a Gal4-AD yeast two-hybrid vector (Figure 6A, bottom) and tested for interaction with BD-Slx5. In the context of full-length AD-Uls1, an internal deletion of amino acids 531–897 was unable to interact with Slx5 (Figure 6B). This defect was not simply the result of an expression or general folding problem, because Uls1Δ531–897 maintained the interaction with BD-Smt3 (SUMO) in the yeast two-hybrid system (Figure 6B) and was expressed well (Figure 6C). To determine whether this region of Uls1 was sufficient for interacting with Slx5, the Uls1_531–897_ fragment and a partially overlapping fragment (Uls1_554-955_) that lacks the purported SIM at amino acids 543–551 (Uzunova *et al.* 2007) were tested in the two-hybrid system. Both the AD-Uls1_531–897_ and AD-Uls1_554–955_ fragments were sufficient for interaction with BD-Slx5 (Figure 6B), and neither interacted with SUMO in the two-hybrid system. Mutations of the Slx5 SIMs that abolish binding to SUMO also had no effect on the Uls1-Slx5 two-hybrid interaction (see Supporting Information, Figure S1). Combined, these results indicated that a region located between the Uls1 SIMs and ATPase domain was required and sufficient to interact with Slx5 *in vivo* and that the Uls1-Slx5 interaction did not require binding of Uls1 or Slx5 to SUMO.

### Domains required for *ULS1* function

**Figure 7:**
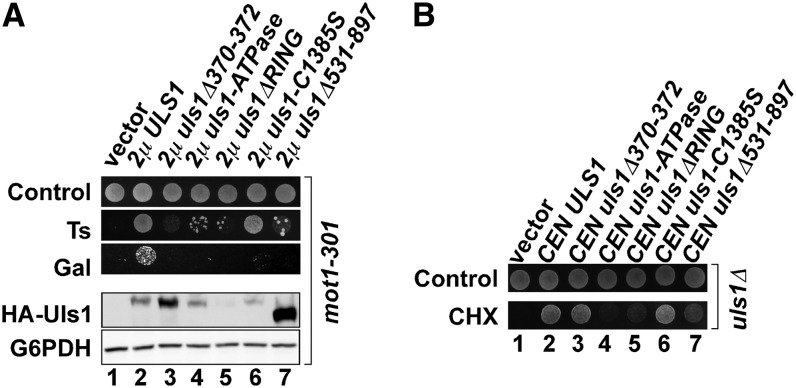
Domains of Uls1 required for its function. (A) 2µ *ULS1* and its indicated derivatives (all tagged with the HA epitope to allow detection of protein expression levels) were introduced into ZY142 (*mot1-301*) and transformants replica plated to test the Ts and Gal phenotypes (upper panel). The *uls1-ATPase* mutant converts both Asp1108 and Glu1109 to Ala (Figure 6A, upper panel). *uls1ΔRING* refers to an internal deletion of the amino acids from 1328 to 1386 in the RING domain. *uls1ΔSIM* refers to an internal deletion of the amino acids from 370 to 372, which removes one of the Uls1 SIMs. Extracts from those strains were prepared, and expression of HA-Uls1 and its derivatives was assayed by Western blotting along with a G6PDH loading control (lower panel). As shown in lane 2, the single HA tag at the N terminus of Uls1 did not affect its function. (B) CEN *HA-ULS1* and its indicated derivatives were introduced into GY2280 (*MOT1^+^ uls1*Δ), and transformants were replica plated to test for complementation of the *uls1*Δ cycloheximide-sensitivity phenotype.

Having identified the region responsible for the interaction with Slx5, we next used two assays to determine the domains of Uls1 that were required for its function. We first tested which of the Uls1 domains was required for its high-copy suppression of *mot1-301*. Missense mutations or deletions (Figure 6A, upper) were generated in the SIMs (Shirai and Mizuta 2008), ATPase, RING, and Slx5-interacting domains that are predicted to inactivate their functions, and those derivatives were tested for their effects on the 2μ *ULS1* plate phenotype (Figure 7A). Mutations in the ATPase, Slx5-interacting domain, and SIMs greatly reduced the *ULS1* high-copy phenotypes (Figure 7A, lanes 3, 4, and 7), with some residual activity observed in the SIM mutant upon further incubation, likely because additional SIM-like motifs have been described for Uls1 (Uzunova *et al.* 2007). The RING domain mutations were less informative because although the *uls1ΔRING* mutation abolishes the suppression phenotype of 2μ *ULS1* (Figure 7A, lane 5), its expression was greatly reduced. The expression of the *uls1-C1385S* RING missense mutation also was reduced compared to that of wild-type *ULS1* but did not abolish the suppression phenotype (Figure 7A, lane 6). Because we and others have been unable to detect E3 activity for Uls1, it remains unknown whether the C1385S mutation abolishes potential E3 activity of Uls1. Taken together, overexpression of *ULS1* suppresses *mot1-301* through a mechanism that minimally requires the Uls1 ATPase and SUMO-binding activities and its interaction with Slx5.

Because a recent large-scale study suggested that *uls1Δ* is sensitive to cycloheximide (Alamgir *et al.* 2010), we tested whether the *uls1* domain mutations complemented the *uls1Δ* cycloheximide-sensitive phenotype when present on a low-copy CEN plasmid. Similar to results obtained for the *ULS1* high-copy phenotype in suppressing *mot1-301*, the Uls1 ATPase domain and the Slx5-interacting region were required to complement the *uls1Δ* cycloheximide-sensitive phenotype (Figure 7B, lanes 4 and 7). The SIM mutation had no effect (Figure 7B, lane 3), and the requirement for the RING domain is difficult to assess (Figure 7B, lanes 5 and 6) for the reasons listed above. These results demonstrated that the ATPase domain and the interaction with Slx5 were important for Uls1 function, both in the context of the *ULS1* overexpression phenotype and in a low-copy number complementation context.

### Biological effect of Uls1–Slx5 interaction

**Figure 8:**
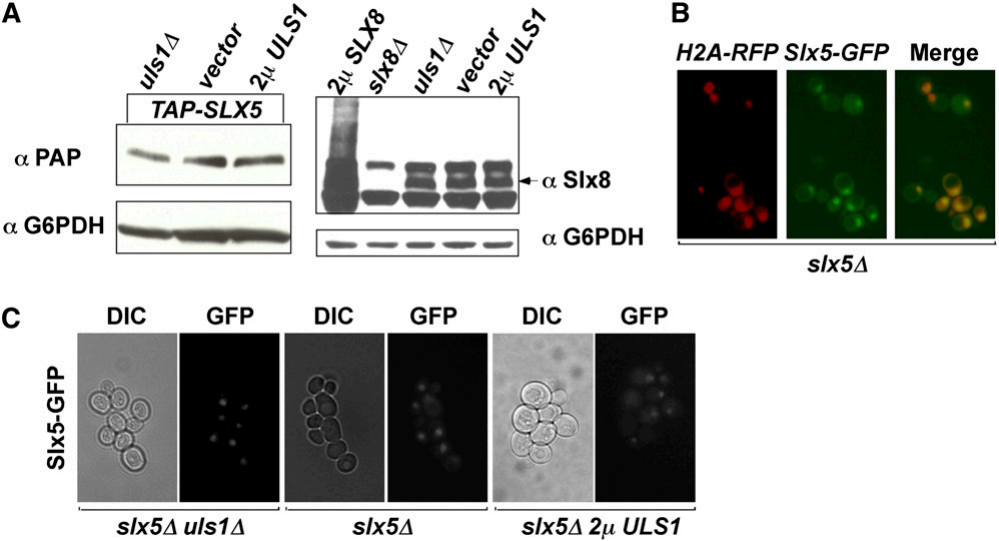
*ULS1* does not affect the protein level of Slx5 or Slx8, or the localization of Slx5. (A) Expression levels of Slx8 and integrated TAP-Slx5 in *uls1*Δ, *ULS1*, and 2µ *ULS1* strains were examined by Western blot with anti-Slx8 and anti-TAP antibodies. (B) Slx5 is localized in the nucleus. CEN *SLX5-GFP* and CEN *H2A-RFP* controls were transformed into an *slx5Δ* strain. Transformants were grown to log phase and examined with microscopy. (C) *ULS1* does not affect the localization of Slx5. CEN *SLX5-GFP* was transformed into the indicated strains. Transformants were grown to log phase and examined with microscopy.

The physical and genetic interactions between *ULS1* and *SLX5* suggest an antagonistic relationship. As shown above, this interaction was important for the function of Uls1, but how does this interaction affect Slx5? It was possible that Uls1 could regulate Slx5 by affecting its expression level, activity, localization, or interaction with other proteins. The protein levels of Slx5 and Slx8 were determined by Western blotting of extracts prepared from *uls1Δ* and 2μ *ULS1* strains, and the localization of Slx5 was examined by expressing *SLX5-GFP* in an *slx5Δ* strain. No changes in the expression of Slx5 or Slx8 were detected (Figure 8A), and Slx5 localized in the nucleus as expected (Chernoff *et al.* 1995), and this localization was not affected by *ULS1* (Figure 8B).

**Figure 9:**
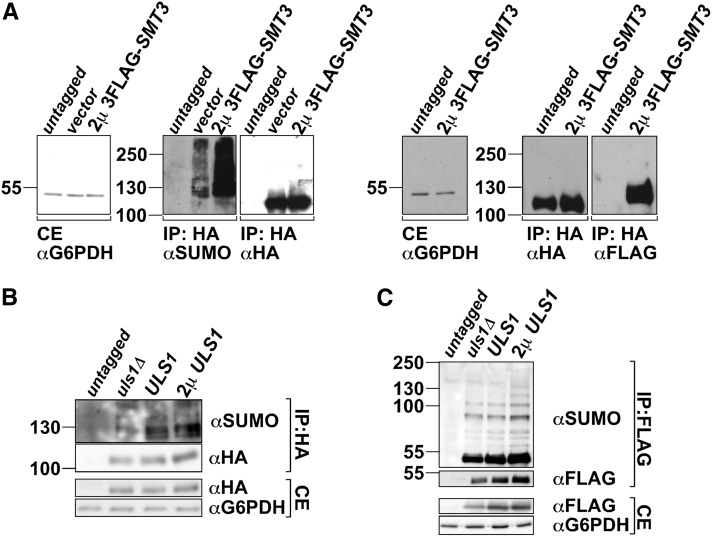
Slx5 is SUMOylated *in vivo* and its SUMOylation decreases in a *uls1*Δ strain. (A) 2µ *SLX5* or 2µ *3HA-SLX5* was overexpressed with vector or 2µ *3FLAG-SMT3* in strain GY285 (*MOT1*^+^) as indicated. 3HA-Slx5 was immunoprecipitated with HA beads (catalog no. A2095; Sigma), and immunoprecipitated samples were subjected to SDS-PAGE and Western blotting (WB) with anti-HA and anti-SUMO antibodies (left panel) or with anti-HA and anti-FLAG antibodies (right panel). (B) 2µ *SLX5* (untagged) or 2µ *3HA-SLX5* was expressed in strains with the indicated genotypes, in the absence of SUMO overexpression. Crude extracts and immunoprecipitated samples were subjected to Western blotting with an anti-SUMO antibody. (C) 2µ *TOA1* (untagged) or 2µ *FLAG-TOA1* was expressed in strains with the indicated genotypes. FLAG-Toa1 was immunoprecipitated with anti-FLAG beads (catalog no. A-2220; Sigma), and immunoprecipitated samples together with crude extracts were subjected to SDS-PAGE and probed with antibodies shown. CE, crude extract.

A recent report (Parker and Ulrich 2012) shows that the SIM- and RING-containing ubiquitin E3 Rad18 is SUMOylated. We therefore tested whether Slx5 was SUMOylated and, if so, whether its modification was affected by *ULS1*. 3HA-tagged Slx5 was immunoprecipitated with HA beads and Western blotted to assess whether it was SUMOylated. When we probed with an anti-SUMO antibody, discrete bands and a high-molecular-weight smear were detected, both of which increased in intensity in a 2μ 3FLAG-*SMT3* strain that overexpresses SUMO (Figure 9A), indicating that Slx5 was SUMOylated. Probing the same samples with an anti-FLAG antibody revealed a band that migrated more slowly than Slx5 when *3FLAG-SMT3* was expressed, confirming that Slx5 was SUMOylated, although the 3FLAG-SUMO fusion was less efficient than untagged SUMO for forming higher molecular weight conjugates. We then examined whether SUMOylation of 3HA-Slx5 was affected by *ULS1*. As shown in Figure 9B, SUMOylation of Slx5 was reduced in the *uls1Δ* strain but not when *ULS1* was overexpressed, indicating that Uls1 affected the SUMOylation of Slx5. To test whether this effect was specific, the SUMOylation of Toa1, a known SUMO substrate, was examined in the *uls1Δ* and 2μ *ULS1* strains (Figure 9C). The Toa1 SUMOylation level remained unchanged regardless of the *ULS1* genotype, indicating that the effect of *uls1Δ* on Slx5 SUMOylation displayed some specificity.

## Discussion

The results presented here reveal unexpected connections between *ULS1* and *SLX5* and provide new insights into their relationship. Previous results suggested that Uls1 and Slx5-Slx8 are independent STUbLs that have overlapping roles in targeting SUMOylated substrates for degradation, based on the presence of SIMs and a RING domain in Uls1, the accumulation of high-molecular-weight SUMO conjugates in a *uls1Δ* strain, the combinatorial phenotypes in *uls1Δ slx5Δ* double mutants, and the physical interactions with ubiquitin E2 Ubc4 detected for both Uls1 and Slx5 (Uzunova *et al.* 2007). Other results, however, suggested that a re-evaluation of the proposed role of Uls1 as a redundant STUbL with Slx5 was warranted. First, no ubiquitin E3 activity has been reported for Uls1 to date. Second, not every RING protein possesses E3 activity; RING proteins such as Slx5, Bard1, Tfb3, and Far1 (Deshaies and Joazeiro 2009) for example, do not have intrinsic ubiquitin E3 activity, although Slx5 and Bard1 associate directly with RING-containing E3s. Third, deletion of genes that do not encode E3s, such as *SGS1*, *SRS2*, and *ULP2*, leads to accumulation of high-molecular-weight SUMO conjugates similar to *uls1Δ* (Mullen and Brill 2008), so the presence of high-molecular-weight conjugates does not necessarily imply loss of a STUbL. Fourth, multiple mutations result in combinatorial growth defects with *slx5Δ* (Pan *et al.* 2006; Wang *et al.* 2006), but many of these genes do not encode ubiquitin E3s (*AOS1*, *SMT3*, *CCR4*, and many others). This is not surprising, as synthetic sick or lethal combinations can be used to infer a functional link, but the mechanistic basis for that link often remains obscure. Finally, it is not known whether the physical interactions of Uls1 and Slx5 with Ubc4 are direct. Thus, in our view the dual issues of whether Uls1 is a STUbL and its relationship with the Slx5-Slx8 STUbL remain open questions.

Because of the inability to express recombinant Uls1, our results do not directly address the issue of whether Uls1 is a STUbL, but they do imply that the relationship between *ULS1* and *SLX5* is more complex than their being STUbLs with partially overlapping functions. First, *ULS1* and *SLX5* displayed opposite patterns of suppression of *mot1-301*; *slx5Δ* strongly suppressed *mot1-301*, but *uls1Δ* had no effect on *mot1-301*, and conversely, overexpression of *ULS1* suppressed *mot1-301*, but overexpression of *SLX5*, *SLX8*, or *SLX5* and *SLX8* did not. These results are most consistent with Uls1 and Slx5-Slx8 having opposing, not overlapping, roles *in vivo*; and because *slx5Δ* but not *uls1Δ* stabilizes Mot1-301, they are not redundant for targeting the ubiquitylation and destruction of Mot1-301. The lack of known substrates is clearly hindering progress with understanding these proteins, but we are not aware of any substrates that are redundantly targeted by Slx5 and Uls1. As additional substrates of Slx5-Slx8 become identified, it will be interesting to test whether the antagonistic relationship that we detect here for Slx5 and Uls1 toward Mot1-301 is also applicable to those substrates. Second, we detected a strong interaction between Slx5 and Uls1 in yeast two-hybrid and co-immunoprecipitation assays, and an internal *ULS1* deletion that specifically abolished the interaction with Slx5 reduced the function of *ULS1* (Figure 7), suggesting that the two proteins function together or that one protein regulates the other. A physical interaction between these two proteins would not be expected if they were acting simply as independent STUbLs. At this point, we cannot distinguish whether the interaction between Uls1 and Slx5 is direct or mediated by another protein that interacts with both Slx5 and Uls1, such as Elg1 (Parnas *et al.* 2011) or Ubc4 (Uzunova *et al.* 2007). Third, increasing expression of *SLX5* reversed the 2μ *ULS1* phenotype (Figure 6A). The simplest interpretation of these combined results is that Uls1 negatively affects the function of Slx5. We expect that Slx5 would be hyperactive in a *uls1Δ* strain, but we cannot assess that prediction due to the absence of any known hypermorphic *SXL5* phenotype.

This study provides the first experimental tests to define the functional domains of Uls1. Mutational analysis confirmed the functional importance of both the ATPase and SIM motifs, and in addition, we were able to identify a domain in Uls1 located between the RING and ATPase domains that was both required and sufficient for interaction with Slx5. Importantly, the interaction-defective mutant *uls1Δ531-897* was defective for the *ULS1* high-copy phenotype and was unable to fully complement the *uls1Δ* phenotype when present on a CEN plasmid, suggesting that the Uls1-Slx5 interaction is functionally relevant. In agreement with the requirement for a separate domain, the SIMs of both Uls1 and Slx5 were not required for the interaction between the two proteins (Figure 6B and Figure S1), excluding the possibility that the interaction is mediated by SUMO or SUMOylated substrates. We cannot rule out the possibility, however, that the SIMs of Slx5 or Uls1 can regulate the interaction.

The genetic links established between *ULS1* and *SLX5* imply that Uls1 inhibits Slx5 and raise the issue of how this might occur mechanistically. Uls1 did not affect the steady-state protein level or cellular localization of Slx5. We therefore suspect that it affects the function of Slx5 at some other level such as its ability to recognize SUMOylated substrates or its interaction with Slx8 or other proteins or by affecting the E3 activity of the Slx5-Slx8 complex. We were able to detect that Slx5 was SUMOylated and that the SUMOylation of Slx5 was reduced by *uls1Δ* (Figure 9B). We attempted to create a SUMOylation-deficient *slx5* mutant to assess its functional significance, but as has been observed for other SUMO substrates (Psakhye and Jentsch 2012), missense mutations at three predicted SUMOylation sites of Slx5 (K31, K465, and K473) either alone or in combination did not abolish the SUMOylation of Slx5 (data not shown). Determining the relevance of SUMOylation on Slx5 function thus will require a more complete study to map and mutate the SUMOylation sites.

Although this report focuses on the relationship between Uls1 and Slx5, two other results from our library screen should prove to be interesting subjects for further study. First, although directed overexpression of *SMT3*, *UBA2*, *UBC9*, and *ULP2*, all of which are known components of the SUMO conjugation and de-conjugation pathway, suppress *mot1-301*, overexpression of the other pathway components *AOS1*, *SIZ1*, *SIZ2*, *ULP1*, and *UBC4* did not suppress. It is unclear whether this is due to a trivial explanation such as the extent of overexpression, or whether it reveals more about the regulation or roles of these genes. Second, the two other high-copy suppressors identified in our screen suppress *mot1-301* by an unanticipated mechanism that is distinct from *ULS1* and the previously identified genomic suppressors (Wang *et al.* 2006). *MOT3* encodes a transcription regulator (Hongay *et al.* 2002; Madison *et al.* 1998) that can exist in a prion state, mediated by its glutamine-rich and asparagine-rich repeats (Alberti *et al.* 2009). *MIT1* also encodes a transcriptional regulator (Cain *et al.* 2012) with an asparagine-rich domain, and it also possesses prion-like properties (Alberti *et al.* 2009). Interestingly, the suppression of *mot1-301* by high-copy *MOT3* and *MIT1*, but not by high-copy *ULS1*, is dependent on *HSP104*, which is required for prion formation and propagation (Chernoff *et al.* 1995; Satpute-Krishnan *et al.* 2007; Wegrzyn *et al.* 2001). This result indicates that the overexpression of *MOT3* and *MIT1* increased the level of the Mot1-301 protein by a different mechanism from overexpression of *ULS1* and that the formation of prions was responsible for their high-copy suppression phenotype. Interestingly, protein aggregates and prions have been reported to inhibit the proteosome (Bence *et al.* 2001; Deriziotis and Tabrizi 2008; Kristiansen *et al.* 2007), and inhibition of proteosomal degradation by *MOT3* and *MIT1* prions provides a satisfying model to explain their effects on stabilization of Mot1-301.

## Supplementary Material

Supporting Information
