# Supplementary material for: Physical and Genetic Interactions Between Uls1 and the Slx5–Slx8 SUMO-Targeted Ubiquitin Ligase
Source: G3 (Bethesda). 2013 Apr 1;3(4):771–80. doi: 10.1534/g3.113.005827 (PMC3618364; doi:10.1534/g3.113.005827)
Supplement: Supporting Information [file supp_3_4_771__index.html]

Physical and Genetic Interactions Between Uls1 and the Slx5–Slx8 SUMO-Targeted Ubiquitin Ligase — Supporting Information 

# Physical and Genetic Interactions Between Uls1 and the Slx5–Slx8 SUMO-Targeted Ubiquitin Ligase

## Supporting Information for Tan, Wang, and Prelich, 2013

**Files in this Data Supplement:**

- Figure S1 - Mutations in the SIMs of Slx5 are not required for the Uls1-Slx5 interaction nor for the complementation of *slx5Δ*. (PDF, 449 KB)
