# Supplementary material for: Physical and Genetic Interactions Between Uls1 and the Slx5–Slx8 SUMO-Targeted Ubiquitin Ligase
Source: G3 (Bethesda). 2013 Apr 1;3(4):771–80. doi: 10.1534/g3.113.005827 (PMC3618364; doi:10.1534/g3.113.005827)
Supplement: Supporting Information [file supp_g3.113.005827_FigureS1.pdf]

G3 Genes | Genomes | Genetics

Physical and Genetic Interactions Between Uls1 and the Slx5-Slx8 SUMO-Targeted Ubiquitin Ligase

Wei Tan[1], Zheng Wang[2], and Gregory Prelich[1*]

1. Department of Genetics, Albert Einstein College of Medicine, Bronx, NY, 10461

2. Current address: The Salk Institute for Biological Studies, La Jolla, CA, 92037

DOI: 10.1534/g3.113.005827

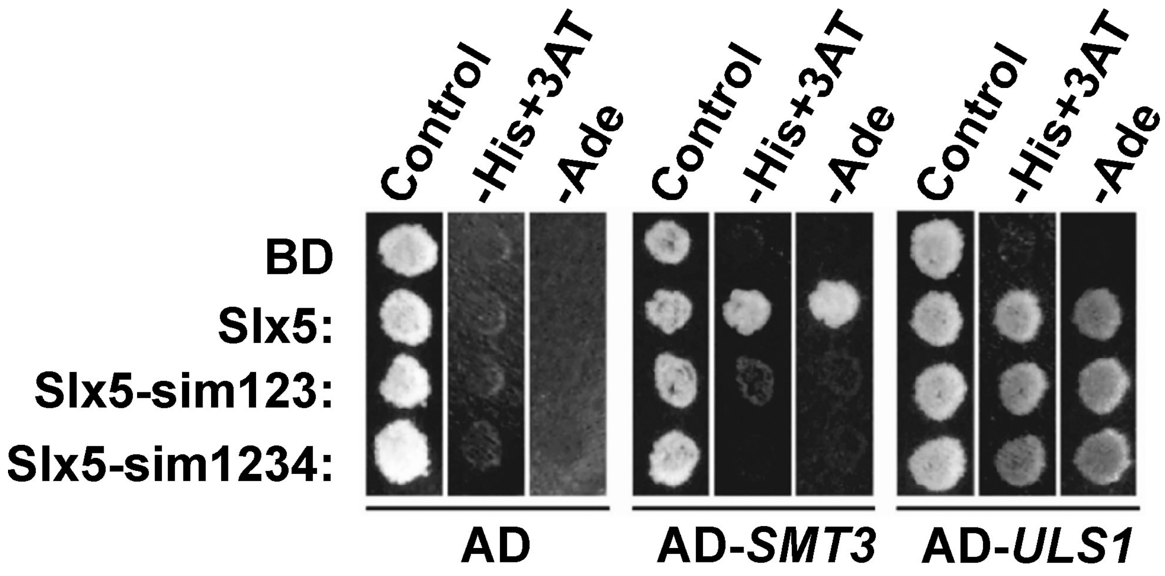

Figure S1 Mutations in the SIMs of Slx5 are not required for the Uls1-Slx5 interaction nor for the complementation of *slx5Δ.* (A) Gal4-BD-*SLX5* and its indicated SUMO interaction motif mutations (Xie *et al.* 2010) were transformed into yeast two-hybrid report strain PJ69-4A with binding domain (AD) only, *AD-SMT3,* or *AD-ULS1*. Transformants were selected and then replica plated to test the indicated phenotypes. 3AT was added to reduce background growth on the SC-His plates.
